# Endoleak detection using single-acquisition split-bolus dual-energy computer tomography (DECT)

**DOI:** 10.1007/s00330-016-4480-6

**Published:** 2016-07-19

**Authors:** D. Javor, A. Wressnegger, S. Unterhumer, K. Kollndorfer, R. Nolz, D. Beitzke, C. Loewe

**Affiliations:** Department of Biomedical Imaging and Image-guided Therapy, Medical University of Vienna, Währingergürtel 18-20, A-1090 Vienna, Austria

**Keywords:** Endoleak, Aorta, Aneurysm, Computed tomography, Angiography

## Abstract

**Objectives:**

To assess a single-phase, dual-energy computed tomography (DECT) with a split-bolus technique and reconstruction of virtual non-enhanced images for the detection of endoleaks after endovascular aneurysm repair (EVAR).

**Methods:**

Fifty patients referred for routine follow-up post-EVAR CT and a history of at least one post-EVAR follow-up CT examination using our standard biphasic (arterial and venous phase) routine protocol (which was used as the reference standard) were included in this prospective trial. An in-patient comparison and an analysis of the split-bolus protocol and the previously used double-phase protocol were performed with regard to differences in diagnostic accuracy, radiation dose, and image quality.

**Results:**

The analysis showed a significant reduction of radiation dose of up to 42 %, using the single-acquisition split-bolus protocol, while maintaining a comparable diagnostic accuracy (primary endoleak detection rate of 96 %). Image quality between the two protocols was comparable and only slightly inferior for the split-bolus scan (2.5 vs. 2.4).

**Conclusions:**

Using the single-acquisition, split-bolus approach allows for a significant dose reduction while maintaining high image quality, resulting in effective endoleak identification.

***Key Points*:**

• *A single-acquisition, split-bolus approach allows for a significant dose reduction*.

• *Endoleak development is the most common complication after endovascular aortic repair (EVAR)*.

• *CT angiography is the imaging modality of choice for aortic aneurysm evaluation*.

## Introduction

Endovascular aneurysm repair (EVAR) is a widely used, minimally invasive technique for the treatment of infrarenal and thoracic aortic aneurysms, and it has become an accepted alternative to open surgery [1, 2]. Endoleak development—defined as persistent periprosthetic flow—is the most common complication after EVAR (rate, 16 % − 33 % [3, 4]) and represents the major risk factor for late rupture of the aneurysm and the main indication for conversion to open repair [5]. It has, therefore, become critical to identify endoleaks that necessitate secondary interventions to ensure long-term success after the procedure and to prevent late rupture. This requires a life-long surveillance strategy with a regular schedule of yearly follow-up examinations.

CT angiography is considered the imaging modality of choice for pre- and postoperative imaging evaluation of abdominal aortic aneurysms. However, there is an on-going debate about the optimal CT acquisition protocol to avoid high cumulative radiation doses [6]. Arterial and venous delayed phases are considered necessary because of the variable flow velocities of endoleaks [7–9]. In addition, unenhanced images are useful in differentiating calcifications in the aneurysm sac from periprosthetic contrast enhancement. Recent studies have shown an increased risk of radiation-induced cancer after repeated exposure from CT scans [10–14], underlining the need for dose-reduction strategies, especially in patients who require repeated CT scanning. Thus, given the need for life-long CT follow-up after abdominal stent-graft placement, the standard CT acquisition protocol after EVAR is currently a trade-off between image quality and radiation exposure.

In this prospective trial, we created a single-phase, dual-energy CT angiography with a split-bolus technique. By injecting intravenous contrast material in two sequential boluses separated by an appropriate time delay, imaging during synchronous arterial and venous delayed phases is possible within one single scan acquisition. If other technical factors are held constant, this approach should reduce the effective radiation dose associated with the CTA examination simply by reducing the number of CT acquisitions obtained.

Furthermore, dual-energy CT provides a wide range of post-processing capabilities for the evaluation of CT angiography data sets [15, 16], and, by taking advantage of one of the main features of dual-energy CT—the possibility to calculate virtual unenhanced images—a further radiation dose reduction should be possible by replacing standard unenhanced images with virtual unenhanced images.

Thus, the aim of this study was to assess whether the image quality and endoleak diagnosis remained unchanged when applying a modified CT acquisition protocol with reduced radiation exposure compared to the previously used standard biphasic protocol (baseline scan).

## Materials and methods

### Patients

Fifty consecutive patients referred to our department to undergo CT angiography after EVAR of abdominal aortic aneurysms were prospectively enrolled in this study. Only patients with a history of at least 6 months post-stent-graft implantation and at least one prior post-EVAR follow-up CT examination at our institution, using the standard biphasic routine protocol (baseline scan), were enrolled (follow-up protocol after EVAR was a CT at 3 days, 6 months, and 12 months, and yearly thereafter). Exclusion criteria included contraindication to intravenous administration of iodine contrast medium, hyperthyroidism, and the presence of renal failure.

After the conclusion of the study, five of the patients were treated because of type II endoleaks with growing aneurysm sacs and type I endoleaks.

The study plan is described in Fig. 2, and the stent-graft devices used for EVAR are listed in Table 1.

**Table 1:** Stent-graft devices used for EVAR

Stentgraft	
Medtronic Endurant II	17
Gore Excluder	13
Cook Zenith	5
Medtronic Talent	3
Trivascular Ovation	2
Bolton Treovance	2
Vascutec Anaconda	2
Vanguard	2
Jotec E-Vita	1
Unknown (due to EVAR in external hospitals)	3

Written informed consent was obtained from all study patients prior to inclusion in the study. The study was approved by the institutional review board (Ek-Nr 2162/2013).

### CT acquisition protocol (standard biphasic baseline scan)

All examinations were performed using a second-generation, dual-source CT scanner (DSCT–SOMATOM Definition Flash, Siemens).

The protocol included an arterial phase acquisition, followed by a venous phase acquisition limited to the extent of the stent graft.

Dual-source MSCTA was performed after the injection of 110 ml Iomeron 400 at an injection rate of 6 ml/s and initiated 15 s after the threshold at the level of the abdominal aorta reached 150 HU (bolus-tracking technique). Venous phase acquisition was initiated 18 s after the arterial phase. The tube voltage was set to 120 kV with a tube current-time product of 120 mAsref/rot.

### CT acquisition protocol (split bolus)

The split-bolus scan was performed in the caudo-cranial direction with the same range as the standard baseline scan protocol using the dual-source CT scanner described above.

The total contrast medium volume was determined as a function of the patient’s BMI, and four subgroups were formed, ranging from 90–130 ml total volume, as illustrated in Table 2.

**Table 2:** Total contrast medium volume and injection flow rates as a function of the patient’s BMI subdivided into four subgroups

	<20 BMI	20-25 BMI	25-30 BMI	>30 BMI
Total contrast medium quantity	90 ml	100 ml	110 ml	130 ml
Contrast medium bolus 1	35 ml 2.3 ml/s	40 ml 2.6 ml/s	45 ml 3 ml/s	55 ml 3.6 ml/s
Contrast medium bolus 2	55 ml 3.6 ml/s	60 ml 4 ml/s	65 ml 4.3 ml/s	75 ml 5 ml/s

The first bolus of contrast medium was administered, based on the body mass index of the patient, followed by a chaser bolus of 20 ml saline solution at a 4 ml/s flow rate. The time between starting the first and the second bolus was 35 s in all the patients. The bolus split ratio was chosen to be 40:60. The reason for the chosen ratio was to make more contrast medium available for the early and clinically more important type I and type III endoleaks.

The scanning delay was determined using a bolus-tracking technique by placing a region of interest (ROI) at the level of the abdominal aorta and simultaneously starting the dynamic monitoring scan. The trigger threshold inside the ROI was set at an absolute value of 130 HU.

A dual-energy scan was initiated 8 s after the attenuation reached the predefined threshold of 130 HU.

The tube voltage for tube A was set to 80 kV, with a tube current-time product of 215 mAsref/rot, and the tube voltage for tube B was 140 kV, with a tube current-time product of 83 mAsref/rot. The chosen parameters ensured a similar CT dose index (CTDI) for the dual-energy acquisition compared to the CTDI of the arterial phase of our baseline scan (CTDIvol = 8.11 mGy). This served to make the DECT scanning protocol radiation-neutral compared to a single-phase arterial scan.

### Data processing and evaluation of the split-bolus scan

**Fig. 1:**
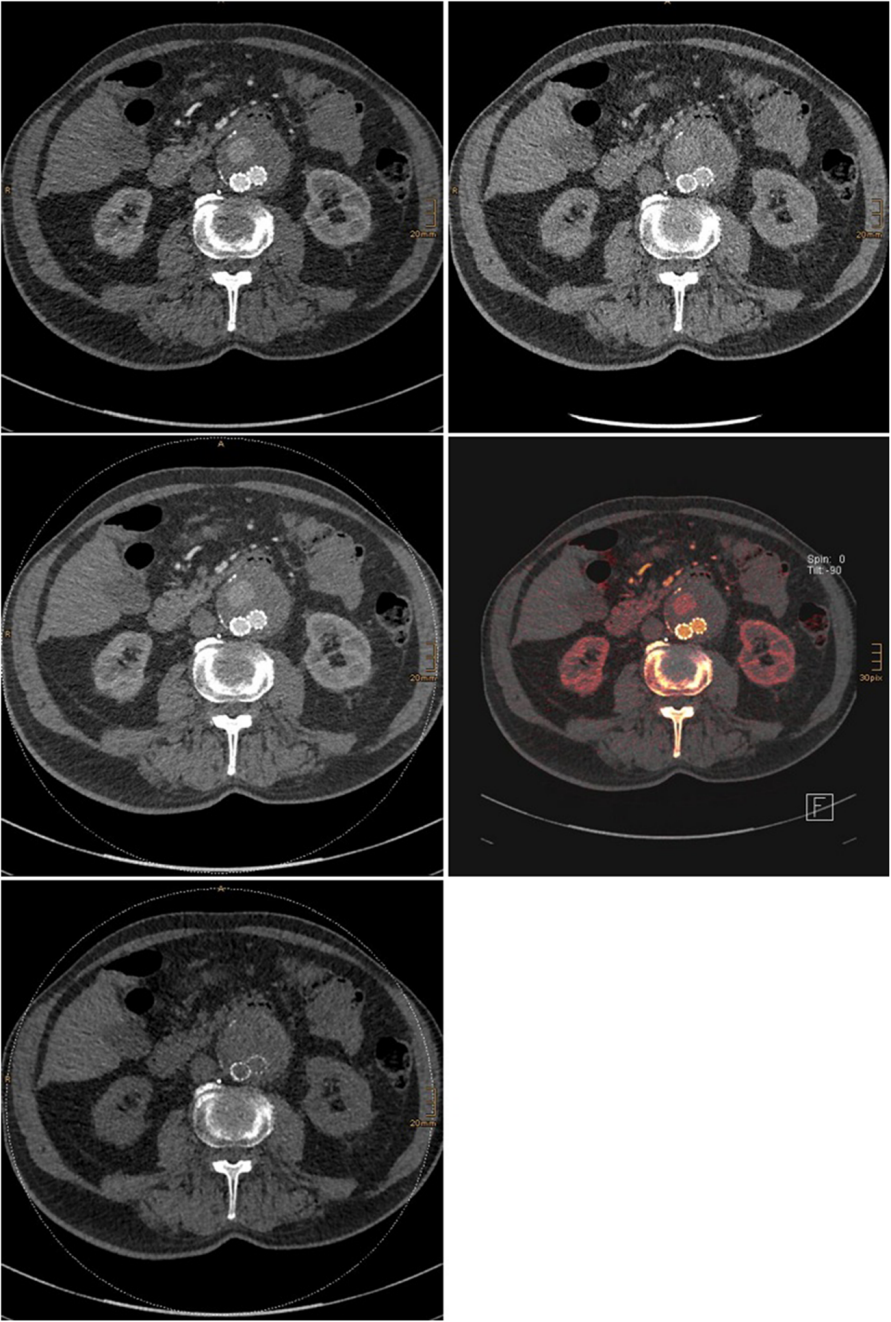
The first three images show the 80-kVp, 140-kVp, and mixed or fused images. Iodine colour-coded (fourth image) and virtual non-contrast (fifth image) CT data sets are also shown

From the raw helical projection data of both tubes in the dual-energy acquisition, three separate data sets were generated: 80 kVp, 140 kVp, and mixed or fused images. From each of these data sets, two series of axial images were then reconstructed. A virtual non-contrast CT data set (VNC) and an iodine colour-coded data set that showed iodine distribution over the virtual non-contrast image were reconstructed transversally and sagittally (Fig. 1).

#### Radiation dose estimation

For the baseline scan acquired using the standard protocol, as well as for the current dual-energy scan, the volume CT dose index [CTDI vol (mGy)] and total dose-length product [DLP (mGy × cm)] were recorded from the CT dose report and compared.

The obtained values of DLP were converted to a corresponding individual effective radiation dose in millisieverts (mSv) by multiplying the DLP by a conversion coefficient [k (mSv/mGy × cm)] (normalised effective dose per DLP over various body regions for adults based on a 32-cm-diameter CT body phantom). The CT scans included the abdomen (k = 0.015 mSv/mGy × cm) and, in some cases, the chest (k = 0.014 mSv/mGy × cm); therefore, the mean of both region-specific conversion coefficients, k = 0.0145 mSv/mGy × cm, was used [17–19].

#### Data Interpretation: reading sessions

The cases were evaluated with regard to endoleak diagnosis and image quality. Two radiologists with 11 and 6 years of experience in body CT angiography, who were blinded to the patient's clinical information and previous imaging findings, evaluated the images.

Axial images, as well as multiplanar reconstructions, were reviewed on external workstations after anonymisation and randomisation.

Two different reading sessions were performed by each reader, separately, and the results were compared subsequently. In cases of disagreement between the two readers, the final decision was made by consensus.

In the first session (session A), the baseline scan was evaluated. The information obtained by the reading of these cases was considered the reference standard for the diagnosis of an endoleak.

In the second session (session B), the readers evaluated the new single-acquisition, dual-energy image data sets. The interval between reading sessions A and B was 3 weeks.

Each case was assessed for the presence of an endoleak. In this evaluation, endoleaks were classified as previously described [20].

Furthermore, the maximum diameter of the aneurysm sac was measured and a potential aneurysm expansion or reduction was assessed. All three image planes (and curved MPRs) were analysed to evaluate the centre line of the aorta. The maximum axial diameter of the aneurysm sac perpendicular to the centre line (longitudinal axis) of the aorta was measured. Aneurysm reduction was defined as a reduction of ≥5 mm in the maximum diameter compared to the previous examination, whereas aneurysm expansion was defined as an increase of ≥5 mm in the maximum diameter. Variations of <5 mm diameter were considered not significant, and the aneurysmal sac was then regarded as stable [21].

The two readers evaluated subjective image quality, using a four-point scale [22], as follows: grade 0 (non-diagnostic); grade 1 (poorly diagnostic); grade 2 (good quality); grade 3 (excellent).

In case of conformity between the two examinations regarding the presence or non-presence of an endoleak, the split-bolus acquisition was considered true positive or true negative (primary agreement rate). An additional requirement was that the diameter of the aneurysm sac was constant or decreasing if no endoleak was present in either examination (single-acquisition DE and biphasic).

In case of a discrepancy between the follow-up examination using the baseline scan and the split-bolus acquisition that could not be explained by the normal clinical course (e.g., newly developed late-onset endoleak and decreasing/constant diameter of the aneurysm sac or disappearance of a previously described endoleak and increasing diameter), an additional MRI examination of the aorta was performed as the final reference standard, and the result was defined as the secondary agreement rate. MRI is currently considered the gold standard for endoleak detection and does not further increase the radiation dose to the patient [23]; furthermore, new unenhanced MRI techniques appear to be promising advancements with regard to endoleak detection [24]. The two readers performed an additional reading session to evaluate the MRI results.

### MRI angiography protocol

The patients were examined on a 1.5-T MR scanner (Magnetom Avanto, Siemens) with a gradient amplitude of 40 mT/m along the x- and y-axes and 45 mT/m along the z-axis and a maximum slew rate of 200 mT/m/ms. A commercially available six-element flexible body coil (Matrix, Siemens Healthcare) was used to image the abdominal aorta.

After the acquisition of a contrast-free mask (coronal T1-weighted gradient-echo sequence) of the whole abdominal aorta and the pelvic vessels, contrast-enhanced, first-pass 3D MRA was performed. A biphasic injection protocol was applied with an automatic power injector. Gadobenate dimeglumine was injected at a flow rate of 2 ml/s, followed by a 21-ml saline flush at a flow rate of 1.2 ml/s. The amount of gadobenate dimeglumine was calculated according to the following formula: 0.2 ml × body weight (kg).

MRA in three phases (arterial, late arterial, and venous) was initiated using a bolus-triggering technique.

Furthermore, a T1 VIBE fat-saturated sequence before and after contrast medium administration was acquired.

## Statistical analysis

Data analysis was performed using the SPSS statistical package (SPSS Windows, version 20.0). Statistical power analysis was performed using the statistical program G*Power (http://www.gpower.hhu.de/). For the detection of small to medium effects (Cohen's d = 0.4) in a two-tailed t-test for paired samples with α = 0.05 and an assumed power (1-β) = 0.80, a total sample size of 50 subjects was calculated. In addition to the descriptive evaluation of the study sample, a paired-samples t-test was performed to compare the radiation parameters between the two protocols. A *p*-value of less than 0.05 (*p* < 0.05) was considered to indicate statistically significant results.

## Results

There was 1 female and 49 male patients; mean age was 75.83 ± 7.44 years. The mean follow-up period between EVAR and the split-bolus examination was 12.32 ± 7.76 months.

Twenty-three endoleaks were found on the prior baseline examination, of which 9 % (*n* = 2) were seen only on the arterial phase, whereas 30 % (*n* = 7) were visible only on the late venous phase. Of the observed endoleaks, 61 % (*n* = 14) were visible on both the arterial and venous phase.

**Fig. 2:**
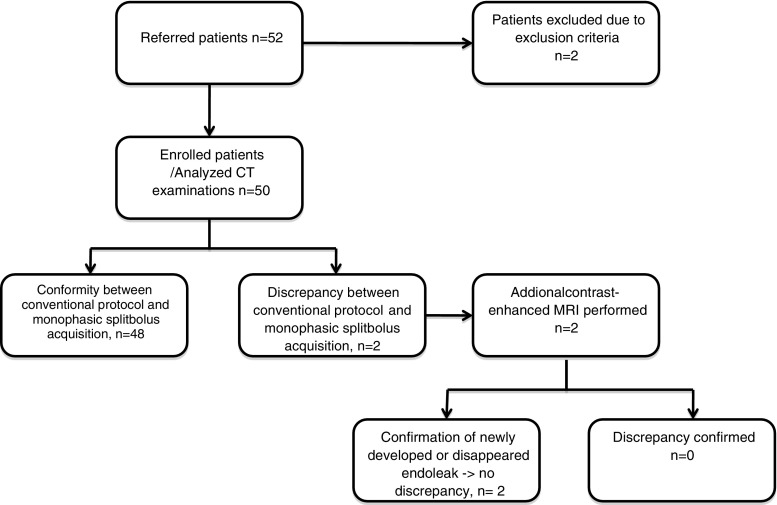
Flowchart of study plan

Twenty endoleaks were found using the split-bolus protocol. One endoleak was seen on the baseline scan, but not on the split-bolus scan. This could be explained by the normal clinical course due to the clearly decreasing diameter of the aneurysm sac. In addition, this result was confirmed by a follow-up CT (using the standard biphasic protocol) after 5 months. Thus, the resulting primary rate of agreement (compared to the baseline examination) was 96 % (*n* = 21), and the rate of non-agreement was 4 %. In those 4 % (*n* = 2) of cases of non-agreement, an additional MRI examination was performed according to the study design. This MRI examination confirmed the split-bolus protocol results in every single case. Therefore, there was a secondary agreement rate of 100 % (Fig. 2).

As expected, most of the observed endoleaks were type II (85 %), two endoleaks (10 %) were type I, and one endoleak was classified as type III (5 %).

The diameter of the aneurysm sac increased in size or remained constant in all cases where an endoleak persisted (endoleak visible on both the standard examination as well as the split-bolus protocol). In all cases of non-existent endoleak (on both examinations), the aneurysm sac decreased in size or remained constant between the two examinations.

The image quality of the contrast-enhanced images of the split-bolus scan was rated either as grade 2 (good) or grade 3 (excellent) in all cases, resulting in an overall grading of 2.4. In contrast, the image quality of the VNC data was inferior and rated as 1.8. The image quality of the baseline scan was rated 2.5 (contrast-enhanced images) and 2.0 (for native images), respectively.

**Fig. 3:**
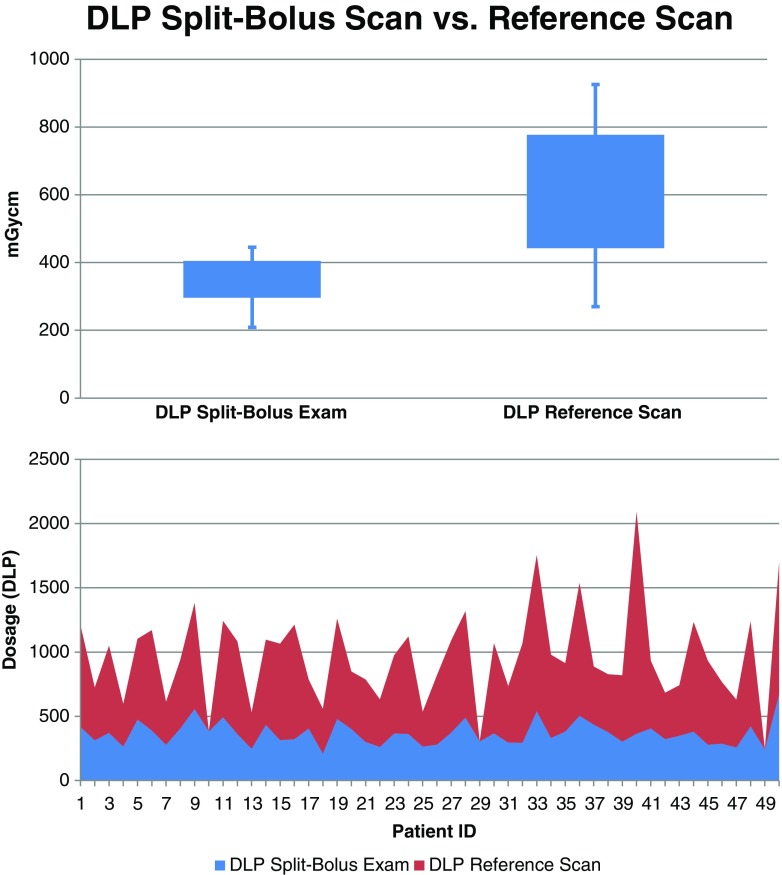
The diagram illustrates the relationship between the dose-length product (mGycm) of the baseline scan and the subsequent split-bolus protocol

A paired-samples t-test was conducted to compare radiation dose parameters between the two test protocols. Results revealed a significant difference in the CDTI for the baseline scan (mean = 15.4, SD = 5.4) and the split-bolus scan (mean = 7.9, SD = 1.7; t(46) = −11.17, *p* < 0.001). A statistically significant difference was also determined for the total dose-length product (DLP). Whereas for the baseline scan a mean DLP of 635.4 mGycm (SD = 270.3) was observed, the mean DLP for the split-bolus scan was 369.9 mGycm [SD =93.0; t(46) = −7.83, *p* < 0.001]. Figure 3 illustrates the relationship of the DLP (mGycm) between the two protocols. The results of this study revealed effective dose values (using a conversion factor of 0.0145) of 9.1 mSv for the baseline scan and 5.3 msV for the split-bolus scan.

## Discussion

A single-acquisition, split-bolus protocol performed on a dual-energy CT scanner was used in this study to test the hypothesis that, compared to the conventional biphasic protocol (baseline scan), the total amount of the radiation dose can be reduced while maintaining a comparable endoleak detection rate. As expected, the radiation dose could be reduced significantly when compared to the baseline scan, while the diagnostic accuracy in detecting endoleaks remained unaffected. The obtained values of DLP and the corresponding individual effective radiation dose showed a reduction of 42 %.

The detection rate for endoleaks using the single-acquisition protocol was sufficient, with only two cases of discrepancy between the two protocols, which seemingly suggested an apparent minimally reduced detection potential. However, all of those unclear cases were reassessed by obtaining an MRI examination, which confirmed the results of the single-acquisition protocol in every single case, resulting in an overall secondary endoleak detection rate of 100 %. The sensitivity for endoleak detection on contrast-enhanced MRI has been described as significantly higher compared to that of CT, and MRI is currently the gold standard for endoleak detection [25, 26]. Based on these findings, the two “discrepant” findings seemed to be attributable more to clinical changes during the follow-up period rather than to differences in the diagnostic accuracy between the two techniques. The measurements of the aneurysmal sac diameter, which decreased (or remained constant) in the case of non-existent endoleaks and increased in cases of endoleak detection, confirmed our results.

There is an on-going debate about the nature of the ideal CT protocol for endoleak detection. With regard to the ideal bolus timing in contrast-enhanced imaging, it remains, for the time being, somewhat unclear whether an arterial or late venous phase is necessary to detect clinically relevant endoleaks, and various studies recommend one or the other. Since cumulative radiation exposure is an important issue in this group of patients, the number of phases acquired is currently a compromise between diagnostic accuracy and radiation dose.

Some authors advocate a monophasic and some suggest a bi- or even tri-phasic protocol. The reason for this inconclusiveness is that endoleaks have variable flow rates, depending on their source, and the blood pool within the aneurysm sac will, therefore, be detectable at variable time points. As a consequence, optimal bolus timing remains a crucial issue in protocol planning. Late ruptures are very rare in cases of type II endoleaks; therefore, several authors recommend only an arterial phase or even only a native scan for routine follow-up; if the diameter remains unchanged, the risk for rupture should be negligible [27].

Some authors advocate the late venous phase after 100 s or even after 300 s, arguing that most endoleaks can be detected [7, 8]. This seems to be true only for type II endoleaks. High-flow type I or type III endoleaks require an arterial phase to be identified [9]. Another role for arterial phase scans is the assessment of aortic branch perfusion (renal, mesenteric, and iliac arteries).

**Fig. 4:**
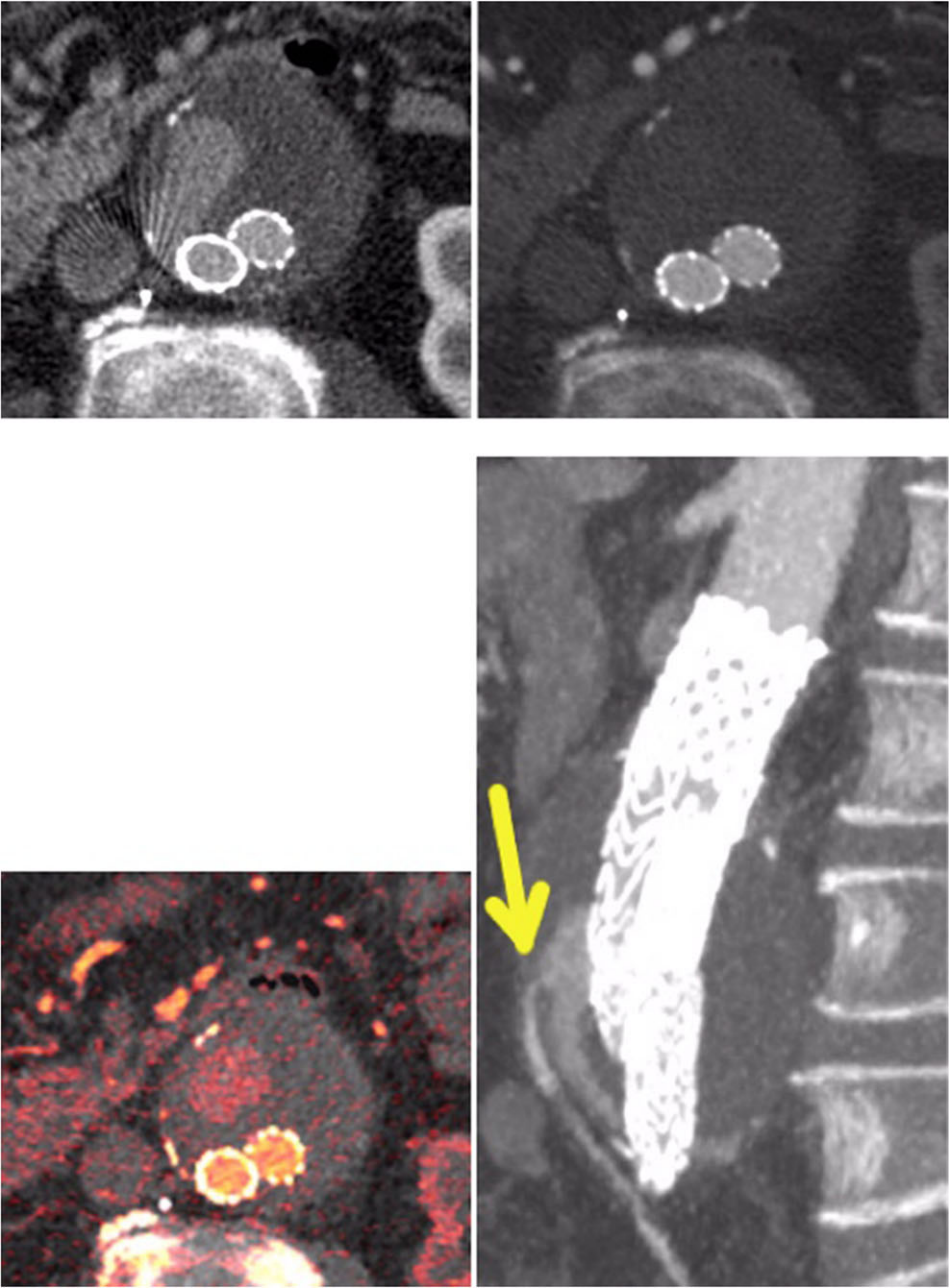
An example of the usefulness of the split-bolus protocol. On the *upper left*, an endoleak can be clearly depicted on the late venous phase, whereas on the arterial phase (*upper right*), the endoleak is hardly visible. The iodine colour-coded data set (*lower left*, corresponding slice) seems to be particularly favourable for depicting endoleaks. On the *lower right* (split-bolus, different patient), the endoleak and the feeding artery (inferior mesenteric artery, *IMA*), are well demarcated

This coincides with our results. Given the large number of type II endoleaks, the evaluation of our results shows that most endoleaks are depictable on both the venous and the arterial phase. However, 30 % of endoleaks were visible exclusively on the venous phase, and two endoleaks (2 %) were seen only on the arterial phase, underlining the need for a combination of arterial and venous phase information for accurate endoleak detection. All of those endoleaks were detectable on the subsequent split-bolus examination (Fig. 4).

We used a split-bolus ratio of 40:60 in order to make more contrast medium available for the early and clinically more important type I and type III endoleaks, but a different ratio of the split bolus could be beneficial, depending on the desired weighting of the arterial or late venous component of the protocol.

**Fig. 5:**
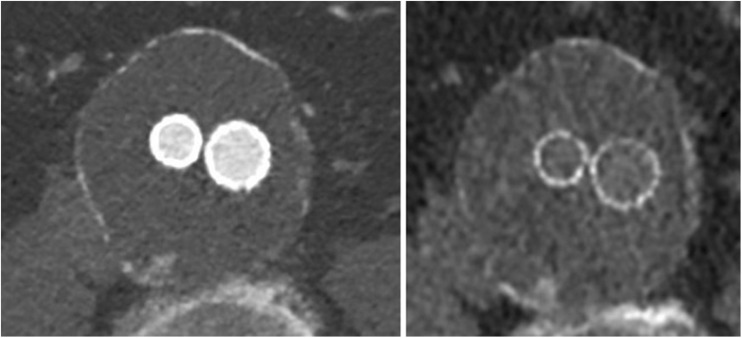
An example of the usefulness of the VNC data set. On the *left*, the contrast-enhanced, split-bolus image shows a questionable type II endoleak, possibly arising from a right lumbar artery. On the *right*, the corresponding slice of the VNC data set reveals a coarse, low-density calcification at that location

One important question is whether non-contrast imaging is necessary. In most cases, calcifications can be distinguished easily from endoleaks on contrast-enhanced CT. However, in a few cases, heavy calcifications, particularly coarse low-density calcifications and haematomas, can mimic endoleaks and make diagnosis difficult. The reconstructed virtual non-contrast (VNC) we used facilitated the differentiation between calcifications and endoleaks significantly (Fig. 5). Nevertheless, we can confirm that, compared to non-contrast scans (native phase), VNC data sets have limitations caused by incorrect calcium subtractions and high noise, which lead to poorer image quality [28, 29].

Recently, promising surveillance strategies that involve contrast-enhanced ultrasound (CEUS) have been proposed [30]. This approach reduces the radiation exposure as well as the contrast medium volume injection. These new protocols consist of CT imaging at 1 month and 1 year after treatment, subsequently followed by CEUS. Nevertheless, the limitations of the method must be considered, such as difficulties of visualisations due to obesity, operator dependency, and artefacts due to echo reflection of graft material in the early follow-up period.

However, this study has several limitations. First, the time interval between the two compared examinations (performed using the conventional and then the split-bolus protocol) was, in some cases, rather long (3 to 12 months) and it could be argued that a late endoleak could have developed in the interval between the examinations that was not detected by the split-bolus protocol. Although this possibility cannot be entirely excluded, the fact that, in those cases, there was no increase in aneurysm sac diameter supports the validity of the data. Second, the number of cases is rather small. However, power analysis revealed a minimum sample size of 50 patients to detect the expected effects. Nevertheless, larger cohorts may provide a more profound knowledge of potential influencing factors, such as age or gender.

The relatively high endoleak rate in our patient sample can be explained by the fact that patients with endoleaks often require more frequent follow-up examinations, especially when the aneurysm sac is growing or after treatment. This could also explain the high incidence rate of type II endoleaks in our consecutive study population.

In conclusion, a dual-energy, split-bolus protocol appears to be a valid radiation-saving alternative for routine follow-up after EVAR. The comparable endoleak detection rate, as well as the significantly reduced radiation dose, supports this approach.
